# Comparative marker analysis of extracellular vesicles in different human cancer types

**DOI:** 10.3402/jev.v2i0.20424

**Published:** 2013-06-18

**Authors:** Yusuke Yoshioka, Yuki Konishi, Nobuyoshi Kosaka, Takeshi Katsuda, Takashi Kato, Takahiro Ochiya

**Affiliations:** 1 Division of Molecular and Cellular Medicine, National Cancer Center Research Institute, Tokyo, Japan; 2 Department of Integrative Bioscience and Biomedical Engineering, Graduate School of Science and Engineering, Waseda University, Tokyo, Japan; 3 Japan Society for the Promotion of Science (JSPS), Tokyo, Japan; 4 R&D Department, SRL Inc., Tokyo, Japan; 5 Department of Biology, School of Education, Waseda University, Tokyo, Japan

**Keywords:** extracellular vesicle marker, prostate cancer cells, breast cancer cells, CD9, CD81

## Abstract

Several cell types, including tumour cells, secrete extracellular vesicles (EVs), and tumour-derived EVs play a role in cancer initiation and progression. These vesicles include both a common set of membrane and cytosolic proteins and origin-specific subsets of proteins that likely correlated to cell type–associated functions. To confirm the presence of EVs in the preparations, researchers have identified so-called EV marker proteins, including the tetraspanin family proteins and such cytosolic proteins as heat shock 70 kDa protein 4 (HSP70) and tumour susceptibility gene 101 (TSG101). However, studies have shown that some EV markers are not always present in all EVs, which not only complicates the identification of EVs but also precludes the quantitative evaluation of EV proteins. Thus, it is strongly required to explore well-conserved EV marker proteins that are present at similar levels, regardless of their tissue or cellular origin. In this study, we compared the presence of 11 well-known EV marker proteins by immunoblotting using EVs isolated from 4 human prostate cell lines and 5 human breast cell lines, including cancer cells with different phenotypes. We found that all the tested EVs were positive for CD9 and CD81, with similar abundance that was irrespective of the EV origin. In contrast, other EV marker proteins, such as TSG101, Rab-5b and CD63, were detected in an inconsistent manner, depending on the origin of the EVs. Thus, we propose that the detection of CD9 and/or CD81 should ensure the presence of EVs.

## Introduction

Extracellular vesicles (EVs), including exosomes, shedding vesicles, prostasomes and apoptotic bodies, are membrane vesicles of 40–1,000 nm that are released from many cell types, including red blood cells, platelets, lymphocytes, dendritic cells, endothelial cells and tumour cells (1–3). These vesicles were classified into 2 types according to their secretory processes. Thus, exosomes are formed in multivesicular endosome, and microvesicles and shedding vesicles originate by direct budding from the plasma membrane (4, 5). Although EV components are different among cells, a certain set of molecules is likely to be commonly contained in EVs, regardless of their origin. These molecules include such proteins as tumour susceptibility gene 101 (TGS101), CD9 and CD63, and some of these proteins are thought to be essential components of EVs. Accordingly, researchers have detected these so-called EV marker proteins by immunoblotting to confirm the presence of EVs (6–18). To our knowledge, however, no studies have performed a comparative analysis using multiple cell lines. Furthermore, recent proteomic studies of EVs have indicated that not all EVs contain these EV marker proteins (according to ExoCarta, a list of the top 25 proteins that are often identified in exosomes; see www.ExoCarta.org) (19–25).

These facts strongly indicate the necessity of reliable EV marker proteins. In this study, we performed a comprehensive comparison of 11 well-known EV marker proteins using 4 prostate cell lines and 5 breast cell lines. We found that CD9 and CD81 were present at similar abundance in the EVs derived from each cell line. In contrast, the abundance of the other tested EV proteins was variable among originating cell types. Thus, we propose that CD9 and CD81 could serve as reliable EV marker proteins.

## Materials and methods

### Cell cultures

The following cells were used: PNT2 cells, an immortalized normal adult prostatic epithelial cell line (DS Pharma Biomedical Co. Ltd., Osaka, Japan); PC3 cells, a human prostate cancer cell line initiated from a bone metastasis of a grade IV prostatic adenocarcinoma (American Type Culture Collection, Manassas, VA); PC-3M-luc cells, a highly metastatic cell line derived from parental PC3 cells (Xenogen, Alameda, CA); 22Rv1 cells, a human prostate cancer cell line, which expresses prostate-specific antigen (PSA) and androgen receptor (American Type Culture Collection); MDA-MB-231-luc-D3H1 cells, which are human breast cancer cell lines (Xenogen); MDA-MB-231-luc-D3H2LN cells, a highly metastatic cell line derived from the parental MDA-MB-231-luc-D3H1 cells (Xenogen); MCF7 cells, a human breast cancer cell line that expresses oestrogen receptor (American Type Culture Collection); and multidrug-resistant MCF7-ADR cells, which originated from parental MCF7 cells (provided by Shien-Lab, Medical Oncology, National Cancer Center Hospital, Tokyo, Japan). The above cells were cultured in RPMI 1640 medium containing 10% heat-inactivated foetal bovine serum (FBS) and an antibiotic-antimycotic (Invitrogen, Grand Island, NY) at 37°C in 5% CO_2_. MCF10A cells, a spontaneously immortalized non-tumourigenic epithelial cell line (American Type Culture Collection), were cultured in MEBM medium with 1% GA-1,000, 50 µg/ml hydrocortisone, 1 µg/ml hEGF, 500 µg/ml insulin and 4% bovine pituitary extract (BPE) (Lonza, Basel, Switzerland) at 37°C in 5% CO_2_.

### Preparation of conditioned medium and EVs

Before the collection of the culture medium, the cells were washed with PBS, and the medium was switched to Advanced RPMI containing an antibiotic-antimycotic and 2 mM l-glutamine (not containing FBS). After incubation for 48 h, the conditioned medium was collected and centrifuged at 2,000× *g* for 10 min at 4°C. To thoroughly remove the cellular debris, the supernatant was filtered through a 0.22 µm filter (Millipore, Billerica, MA). The conditioned medium (CM) was then used for EV isolation.

For EV preparation, the CM was ultracentrifuged at 110,000× *g* for 70 min at 4°C. The pellets were washed with 11 ml of PBS by ultracentrifugation at 110,000× *g* for 70 min at 4°C and resuspended in PBS. The putative EV fraction was measured for its protein content using a Quant-iT™ Protein Assay with Qubit^®^2.0 Fluorometer (Invitrogen).

### Preparation of cell lysates

Whole cell lysates were prepared with Mammalian Protein Extract Reagent (M-PER; Thermo Scientific, Rockford, IL). After the collection of the conditioned medium, cells in a 150 mm culture dish were washed in PBS and then 2 ml M-PER was added. Whole cell lysates were transferred to a 1.5 ml tube and then treated with sonication.

### Reagents

The following antibodies used for immunoblotting were purchased from BD Biosciences (San Jose, CA): mouse monoclonal anti-human CD63 (clone H5C6, dilution 1:200), mouse monoclonal anti-Hsp70 (clone 7/HSP70, dilution 1:1,000), mouse monoclonal anti-Flotillin-1 (clone 18/Flotillin-1, dilution 1:500), mouse monoclonal anti-Caveolin 1 (clone 2297/Caveolin 1, dilution 1:1,000) and mouse monoclonal anti-cytochrome *c* (clone 7H8.2C12, dilution 1:500). Mouse monoclonal anti-human Tsg101 (clone 4A10, dilution 1:1,000) was purchased from Gene Tex (San Antonio, TX). Mouse monoclonal anti-human CD9 (clone ALB 6, dilution 1:200), mouse monoclonal anti-human CD81 (clone 5A6, dilution 1:200), rabbit polyclonal anti-Rab-5b (A-20, dilution 1:200) and mouse monoclonal anti-Annexin 2 (clone C-10, dilution 1:200) were purchased from Santa Cruz Biotechnology (Santa Cruz, CA). Mouse monoclonal anti-human Integrin beta 1 (clone 12G10, dilution 1:1,000) was purchased from Abcam (Cambridge, UK). Mouse monoclonal anti-Actin (clone C4, dilution 1:1,000) was purchased from Millipore. Two secondary antibodies (horseradish peroxidase–labelled sheep anti-mouse and donkey anti-rabbit antibodies) were purchased from GE HealthCare (Little Chalfont, UK).

### Immunoblotting

Equal amounts of EVs or whole cell lysates were loaded onto 4–15% Mini-PROTEAN TGX™ gels (Bio-Rad, Munich, Germany). Following electrophoresis (100 V, 30 mA), the proteins were transferred to a polyvinylidene difluoride membrane. The membranes were blocked with Blocking One solution (Nacalai Tesque, Kyoto, Japan) and then incubated with primary antibodies. After washing, the membrane was incubated with horseradish peroxidase–conjugated sheep anti-mouse IgG or donkey anti-rabbit IgG, and then subjected to enhanced chemiluminescence using ImmunoStar LD (Wako, Osaka, Japan). CD63, CD9, CD81 and Integrin beta 1 were detected under non-reducing conditions.

### Phase-contrast transmission electron microscopy

The isolated EVs were visualized by Terabase Inc. (Okazaki, Japan) using phase-contrast transmission electron microscopy, which can generate high-contrast images of nanostructures of soft materials, including such biological samples as liposomes, viruses, bacteria and cells, without staining processes that may cause damage to the samples. The natural structure of the sample distributed in solution can be observed by preparing the sample using a rapid vitreous ice-embedding method and using cryo–phase-contrast transmission electron microscopy.

### Measurement of size distribution by nanoparticle tracking analysis

Nanoparticle tracking analysis (NTA) was carried out using the Nanosight LM10HS with a blue laser system (NanoSight, Amesbury, UK) on isolated EVs diluted 500-fold with PBS for analysis. The system focuses a laser beam through a suspension of the particles of interest. These are visualized by light scattering using a conventional optical microscope aligned perpendicularly to the beam axis, which collects light scattered from every particle in the field of view. A 60 s video recorded all events for further analysis by NTA software. The Brownian motion of each particle was tracked between frames to calculate its size using the Stokes–Einstein equation.

## Results and discussion

### Validation of EV protein markers by immunoblotting

**Fig. 1:**
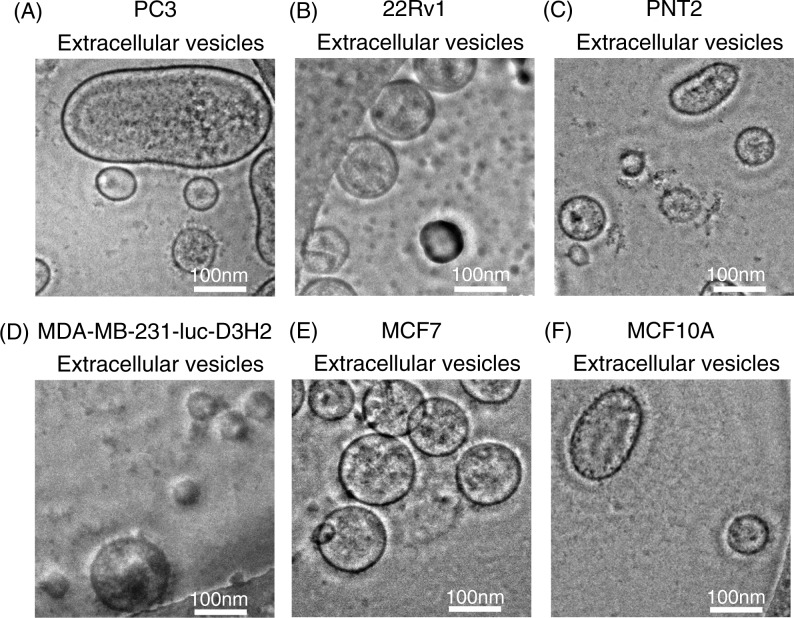
Morphology of purified EVs from 6 cell lines, including cancer cells and non-cancer cells. Shown are representative phase-contrast transmission electron microscopy images of EVs from (A) PC3 cells, (B) 22Rv1 cells, (C) PNT2 cells, (D) MDA-MB-231-luc-D3H2LN cells, (E) MCF7 cells and (F) MCF10A cells. The EVs were purified from the culture supernatants of these cells using a conventional centrifugation method. The scale bar indicates 100 nm.

**Fig. 2:**
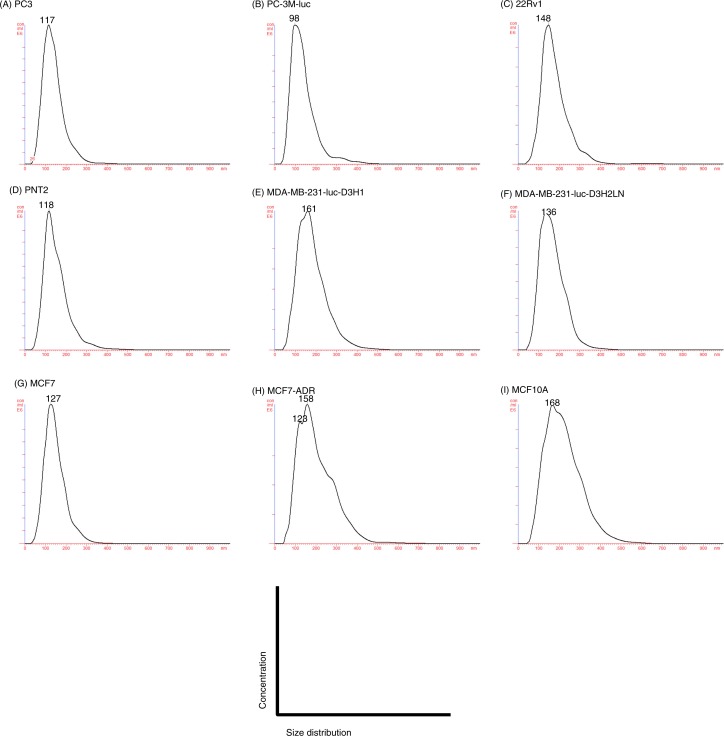
Analysis of the size distribution in the EVs derived from each cell line by the NanoSight particle tracking system. (A) PC3 cells, (B) PC-3M-luc cells, (C) 22Rv1 cells, (D) PNT2 cells, (E) MDA-MB-231-luc-D3H1 cells, (F) MDA-MB-231-luc-D3H2LN cells, (G) MCF7 cells, (H) MCF7-ADR cells and (I) MCF10A cells. The EVs were purified from the culture supernatants of these cells using a conventional centrifugation method. Data from each measurement are shown, revealing the overall size distribution (histograms) and mode (nm).

EVs were purified by ultracentrifugation from conditioned media collected from a variety of prostate and breast cell lines, including cancer and non-cancer cell lines (Table I). After the collection of EVs, we confirmed their size and morphology by phase-contrast transmission electron microscopy (Fig. 1) and the Nanosight particle tracking system (Fig. 2). We observed typical bilayered-membrane vesicles that were heterogeneous in size, ranging in diameter from 50 to 400 nm in the 110,000 × *g* pellets obtained from 6 cell lines, including cancer cells and non-cancer cells (Fig. 1). Furthermore, the size of all the tested EV preparations measured by the nanosight particle tracking system showed a peak between 100 and 200 nm (Fig. 2).

**Table I:** List of cell lines

Cell line	Organ	Disease	Morphology	Additional description
PC3	Prostate	Adenocarcinoma	Epithelial	Tumor stage: grade IV; derived from metastatic site: bone, androgen hormone insensitive
PC-3M-luc	Prostate	Adenocarcinoma	Epithelial	Derived from parental PC3 cells, highly metastatic cell line
22Rv1	Prostate	Carcinoma	Epithelial	Expression of androgen receptor and PSA
PNT2	Prostate	–	Epithelial	Immortalization by transfection with a plasmid containing SV40 genome
MDA-MB-231-luc-D3H1	Mammary gland	Adenocarcinoma	Epithelial	Derived from metastatic site: pleural effusion; receptor: EGF, TGFα
MDA-MB-231-luc-D3H2LN	Mammary gland	Adenocarcinoma	Epithelial	Derived from parental MDA-MB-231, highly metastatic cell line
MCF7	Mammary gland	Adenocarcinoma	Epithelial	Derived from metastatic site: pleural effusion; receptor: estrogen receptor
MCF7-ADR	Mammary gland	Adenocarcinoma	Epithelial	Derived from parental MCF7 cells, docetaxel-resistant cells
MCF10A	Mammary gland	Fibrocystic disease	Epithelial	Produced by long-term culture in serum free medium with low Ca^2+^ concentration.

Note: –, PNT2 cell line was established from normal adult prostatic epithelial cells.

**Fig. 3:**
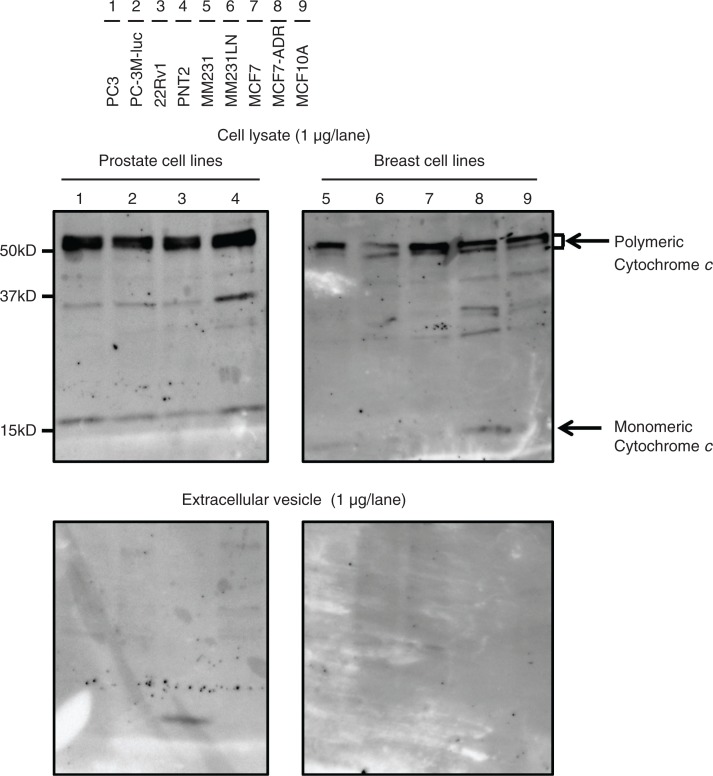
Immunoblotting analysis for cytochrome *c*, a negative control of EVs. Proteins from whole cell lysates (upper panel) or EVs (lower panel) were separated on SDS-PAGE gels followed by Western blotting using antibodies against cytochrome *c*. A 1 µg sample of cell lysate and 1 µg of EV proteins were used. Lane 1: PC3 cells; lane 2: PC-3M-luc cells; lane 3: 22Rv1 cells; lane 4: PNT2 cells; lane 5: MDA-MB-231-luc-D3H1 cells (MM231); lane 6: MDA-MB-231-luc-D3H2LN cells (MM231LN); lane 7: MCF7 cells; lane 8: MCF7-ADR cells and lane 9: MCF10A cells.

**Fig. 4:**
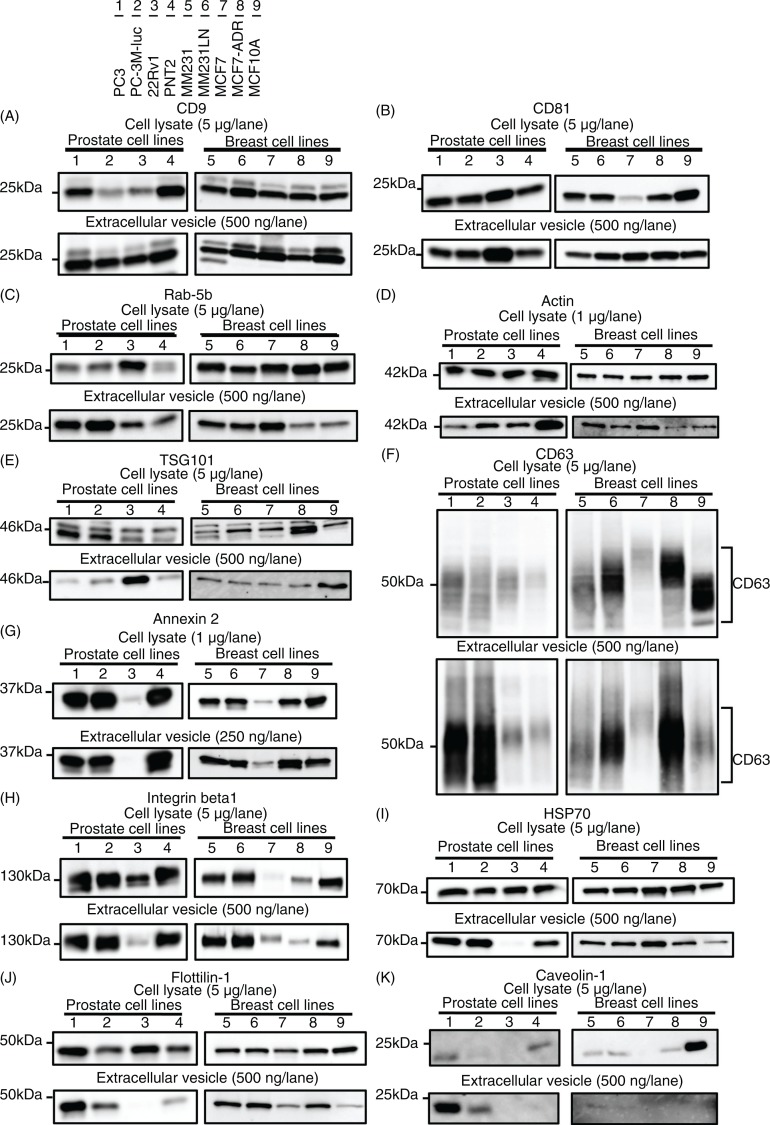
Immunoblotting analysis for conventional markers of EVs derived from prostate cell lines and breast cell lines. Proteins from whole cell lysates (upper panel) or EVs (lower panel) were separated on SDS-PAGE gels, followed by Western blotting using antibodies against 11 different EV markers. A 1 µg sample of cell lysate was used for the detection of Annexin 2 and actin. The other markers were detected using 5 µg of whole cell lysate. A 250 ng sample of EV proteins was used for the detection of Annexin 2. The other markers were detected using 500 ng of EV proteins. CD63, CD9, CD81 and Integrin beta 1 were detected under non-reducing conditions. Lane 1: PC3 cells; lane 2: PC-3M-luc cells; lane 3: 22Rv1 cells; lane 4: PNT2 cells; lane 5: MDA-MB-231-luc-D3H1 cells (MM231); lane 6: MDA-MB-231-luc-D3H2LN cells (MM231LN); lane 7: MCF7 cells; lane 8: MCF7-ADR cells and lane 9: MCF10A cells.

Immunoblotting was performed to compare the abundance of 11 well-known EV markers for these cells and EVs of various origins (Table I), with the immunoblotting of mitochondrial proteins cytochrome *c* performed for quality control prior to this experiment. Cytochrome *c* was readily detectable in the whole cell lysates, but it was completely absent in the EV samples (Fig. 3), indicating that the EV preparations were not contaminated with cellular debris. Thus, we proceeded to the comparative analyses of EV marker proteins using these samples. We summarize the obtained results (Fig. 4) into the following 4 points.

First, and most importantly, CD9 and CD81 were highly enriched in all the tested EV preparations, regardless of their origins (Fig. 4A and B). It should be noted that the abundance of CD9 and CD81 in the EVs from each cell line was comparable, whereas that in their parent cells varied among the cell types. This result strongly suggests the relevance of CD9 and CD81 as loading controls in the analysis of EV proteins.

Second, in contrast to CD9 and CD81, Ras-related protein Rab-5b, cytoskeletal protein actin, TSG101 and CD63 were detected in all EV preparations, but their abundance varied according to their origin (Fig. 4C–F). These results indicate that these molecules can be used to confirm the presence of EVs, as has been proposed in many reports. However, the variable EV abundance of these molecules underscores their limited ability as a loading control.

Third, the EV levels of phospholipid- and RNA-binding protein Annexin 2 and adhesion molecule Integrin beta-1 mirrored the cellular expression levels (Fig. 4G and H). For instance, Annexin 2 was barely detectable as both an EV and cellular protein from 22Rv1 and MCF7 cells. Similarly, Integrin beta-1 was slightly detectable as an EV and cellular protein from 22Rv1, MCF7 and MCF7-ADR cells. These results indicated that the EV abundance of certain proteins was dependent on their expression level in the parent cells.

Fourth, the expression profiles of certain EV proteins did not reflect those of the parent cells. Although heat shock protein HSP70 and raft-associated protein flottilin-1 were detected in each cell line at similar levels, these proteins were barely detected in the EVs derived from 22Rv1 cells (Fig. 4I and J). Additionally, although TSG101 was expressed by each tested cell line, it was highly enriched exclusively in the 22Rv1 cell-derived EVs (Fig. 4E). These observations conflict with the general understanding that the expression profile of a protein in cells is positively correlated with that of the produced EVs (26–28). An explanation can be given for this paradox, considering that the biogenesis pathways of EVs or sorting mechanisms of proteins may be different among these cell lines. Indeed, clearly distinct expression levels were observed for a multifunctional raft-associated membrane protein, Caveolin-1, a protein related to vesicle formation, at both the cellular and EV levels (Fig. 4K). Although Caveolin-1 was detected exclusively for MCF10A cells at the cellular level, its detection at the EV level was limited to PC3 and PC-3M-luc cells. In accordance with the previous report that the prostasomes derived from PC3 cells contain Caveolin-1 (29), we detected Caveolin-1 in the EVs derived from PC3 and PC-3M-luc cells. However, we do not have an explanation for its selective expression at the cellular level. These results again highlight the importance of the identification of well-conserved EV markers that exhibit similar abundance, regardless of their cellular origin or biogenesis pathway.

Unlike CD9 and CD81, our results revealed that the EV abundance of CD63, another well-known and often-employed tetraspanin EV marker, was variable depending on the cell types. One explanation for the different abundance of these tetraspanin EV markers can be given by considering the relationship of these molecules to cancer malignancy. Figure 4 shows that the expression level of CD63 was extremely high in the malignant cancer cell lines, such as PC3, PC-3M-luc, MDA-MB-231-luc-D3H1 and MDA-MB-231-luc-D3H2LN, which have high metastatic ability (Table I), and MCF7-ADR, which has high drug resistance ability compared with its parental cell line MCF7 (Table I). Moreover, the abundance of CD63 was low in the EVs derived from the non-cancer cell lines PNT2 and MCF10A. It has been reported that CD63 interacts with tissue metalloprotease inhibitor protein-1 (TIMP-1) at the cell surface in breast cancer epithelial cells, thereby facilitating its interaction with β1 integrins, resulting in cell survival signalling and the inhibition of apoptosis (30). Concomitantly, CD63 knockdown reduces the binding of TIMP-1 to the cell surface and its interaction with β1 integrins (30). In addition, a correlation between decreased CD63 expression and increased malignancy has also been observed in many other tumours (31, 32). These reports and our results from several prostate and breast cell lines call attention to the use of CD63 as an EV marker, particularly in cancer research.

In summary, we found that the tetraspanin family proteins CD9 and CD81 were highly enriched at similar levels in the EVs derived from all the examined cell lines (Table II). Furthermore, we found that the accumulation of CD9 and CD81 was higher than that of other proteins in EVs (Supplemental Table I). Most importantly, the amount of CD9 and CD81 was generally conserved among the tested cell lines, even those showing different metastatic ability or drug resistance. In contrast, other conventionally used EV markers exhibited distinct abundance according to the originating cell type. Although further comprehensive studies may be required, the present study provides important insight into the field of EV research. In conclusion, we propose that CD9 and CD81 should be useful as marker proteins for the presence of EVs and that these molecules would further allow the quantitative analyses of EV proteins.

**Table II:** Summary of immunoblotting for EV marker proteins

	cell line/marker	CD9	CD81	Rab-5b	Actin	TSG101	CD63	Annexin 2	Integrin beta 1	HSP70	Flottilin-1	Cavolin-1
Prostate	PC3	+++	++	+++	+	+	+++	+++	+++	+++	+++	+++
	PC-3M–luc	+++	++	+++	++	+	+++	+++	+++	+++	++	+
	22Rv1	+++	+++	++	++	+++	+	−	−	−	−	−
	PNT2	+++	++	+	+++	+	+	+++	+++	++	+	−
Breast	MM231	++	++	++	++	+	++	+++	+++	++	++	−
	MM231LN	+++	+++	++	++	+	++	++	++	++	++	−
	MCF7	+++	+++	+++	++	+	+	+	+	+++	+	−
	MCF7-ADR	++	+++	+	+	+	+++	+++	+	++	++	−
	MCF10A	+++	+++	+	+	++	+	++	++	+	+	−

Note: −, not detectable; +, weakly detectable; ++, normally detectable; + ++, strongly detectable. MM231, MDA-MB-231-luc-D3H1; MM231LN, MDA-MB-231-luc-D3H2LN.
